# Supplementary material for: Diverging importance of drought stress for maize and winter wheat in Europe
Source: Nat Commun. 2018 Oct 12;9:4249. doi: 10.1038/s41467-018-06525-2 (PMC6185965; doi:10.1038/s41467-018-06525-2)
Supplement: Supplementary file 1 — Supplementary Information [file 41467_2018_6525_MOESM1_ESM.pdf]

Diverging importance of drought stress for maize and winter wheat in Europe

Webber *et al*

Supplemental Information

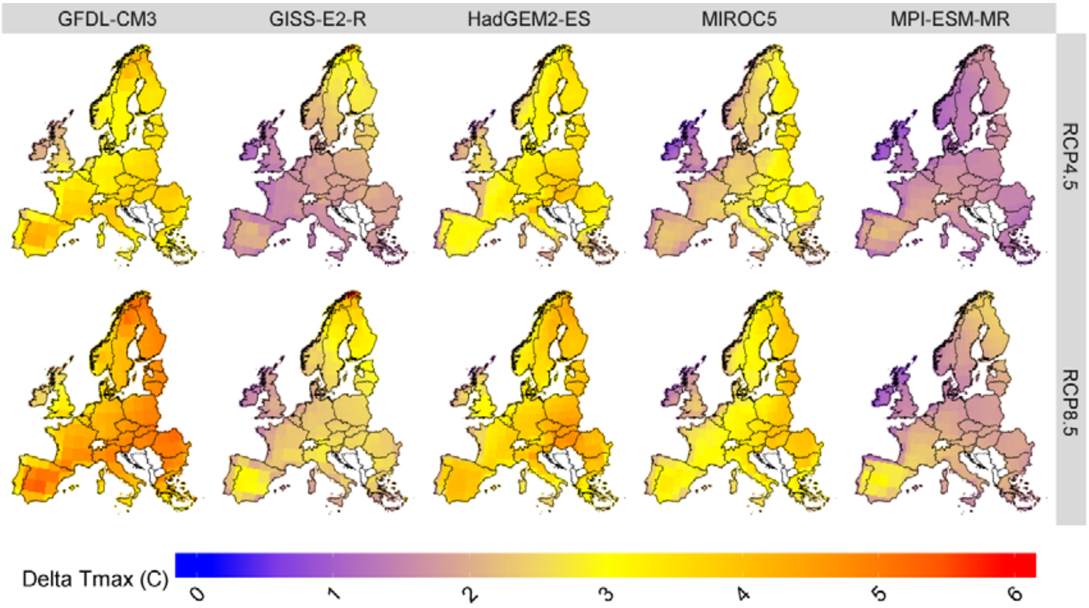

Supplementary Figure 1. Change in daily maximum temperature to 2055. *Absolute change in average daily maximum temperature (°C) between the 2055 scenario period (2040-2069) and the baseline period (1980 to 2010) for RCPs 4.5 and 8.5 for five GCMs: GFDL-CM3, GISS-ES-R, HadGEM2-ES, MIROC5 and MPI-ESM-MR.*

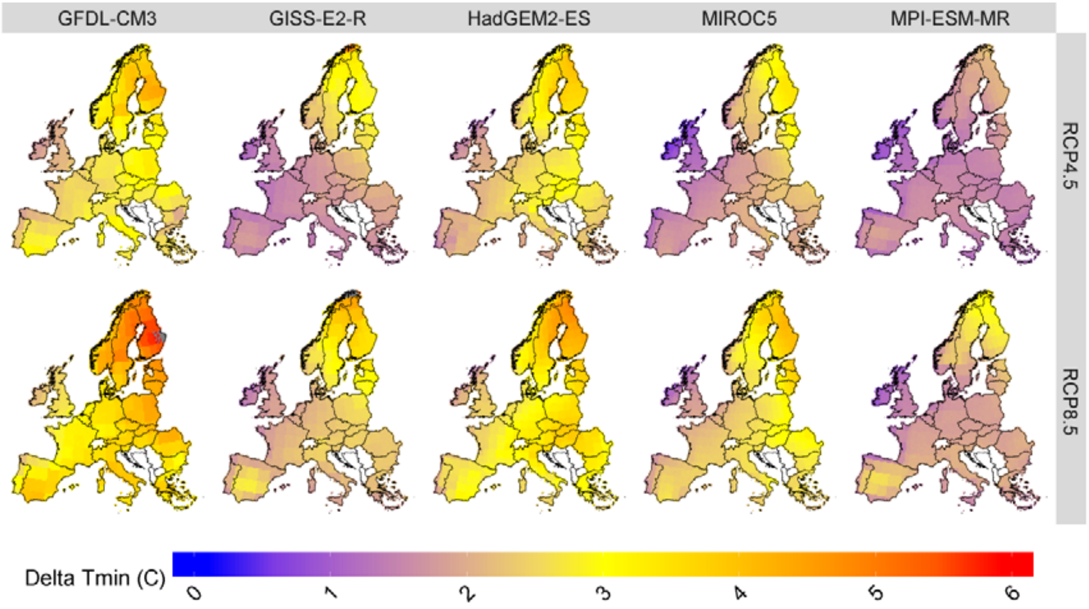

Supplementary Figure 2. Change in daily minimum temperature to 2055. *Absolute change in average daily minimum temperature (°C) between the 2055 scenario period (2040-2069) and the baseline period (1980 to 2010) for RCPs 4.5 and 8.5 for five GCMs: GFDL-CM3, GISS-ES-R, HadGEM2-ES, MIROC5 and MPI-ESM-MR.*

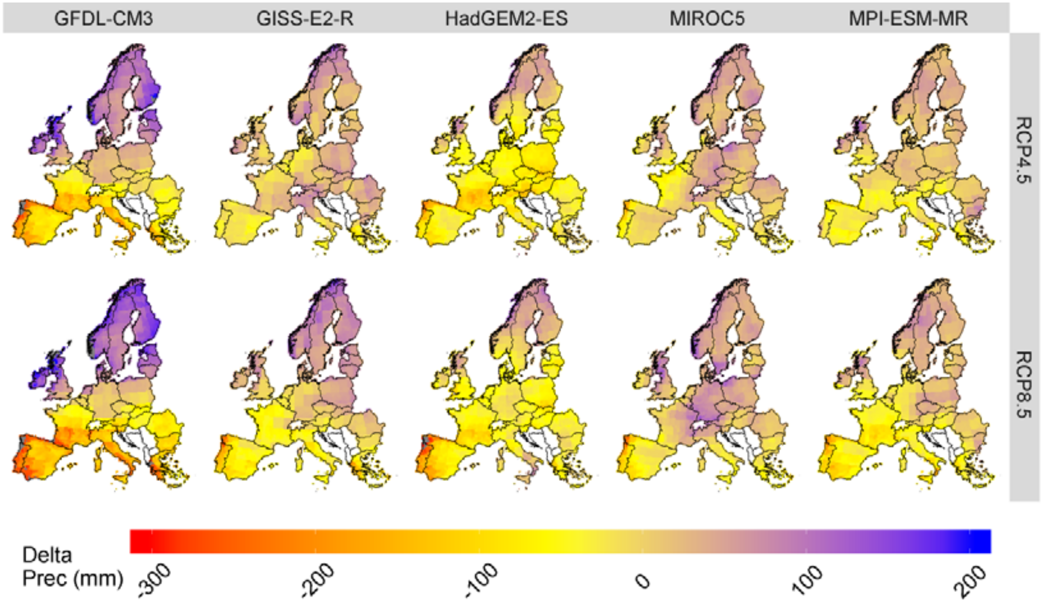

***Supplementary Figure* 3. Change in annual precipitation sum to 2055**. *Absolute change in annual precipitation (mm) for RCPs 4.5 and RCP8.5 between the 2055 scenario period (2040-2069) and the baseline period (1980 to 2010) for five GCMs: GFDL-CM3, GISS-ES-R, HadGEM2-ES, MIROC5 and MPI-ESM-MR.*

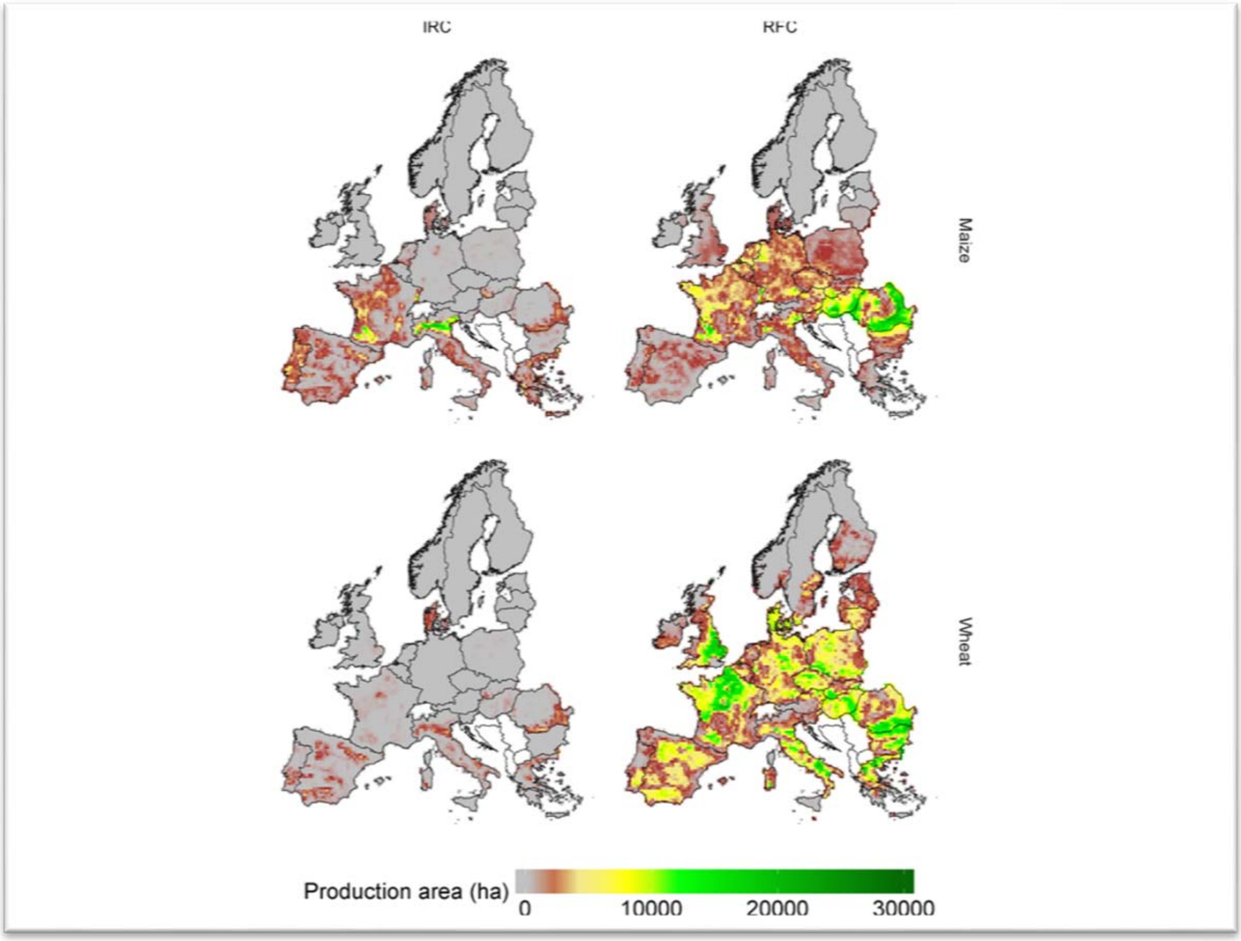

***Supplementary Figure 4. The production area (ha) for irrigated (IRC, left column) and rainfed (RFC, right column) maize (top row) and winter wheat (bottom row) from the MIRCA2000 database (*https://www.uni-frankfurt.de/45218031/data_download*).** Areas shaded in grey have no reported production of the respective crop.*

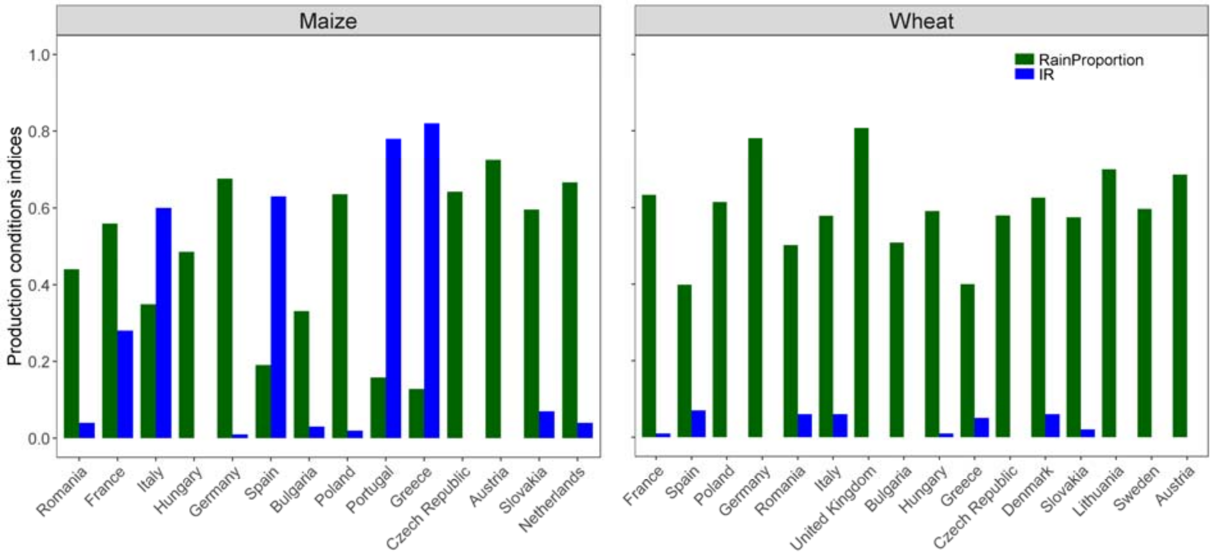

Supplementary Figure 5. National characterization of crop production aridity and prevalence of irrigation. *Green bars indicate the model median's average ratio of rainfed to irrigated yields at the country level for the period 1980 to 2010. Blue bars indicate the percentage of crop land under irrigation in each country (IR).*

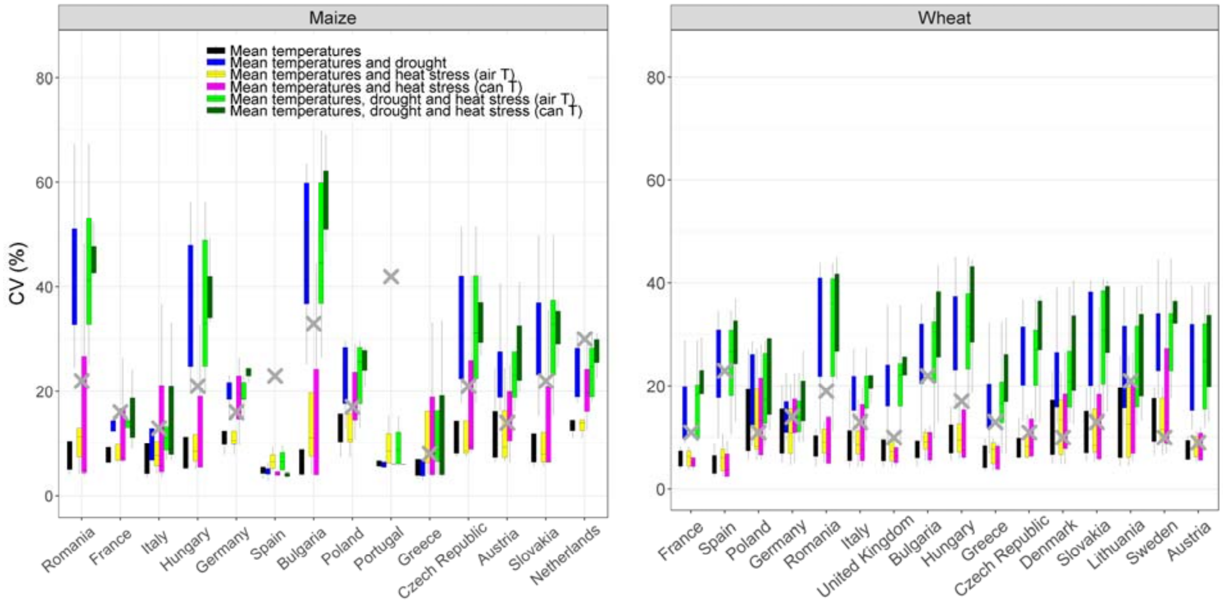

Supplementary Figure 6. The coefficient of variation (CV) of national yields*. CV was calculated between 1984 and 2009 are shown for the FAO-stat observations (grey x symbol) and with the boxplots for the six simulation sets (black- optimal temperature effects only, blue – mean temperature and drought effects, yellow – mean temperature and high air-temperature effects, magenta - mean temperature and high canopy-temperature effects, light green - mean temperature, drought and high air-temperature effects, and dark green - mean temperature, drought and high canopy-temperature effects). The boxplots indicate the uncertainty the crop model ensemble. Countries are listed if they contain more than 1% of the European production area for that crop and are listed from largest area to smallest. For each crop, countries are ordered by production area in descending order.*

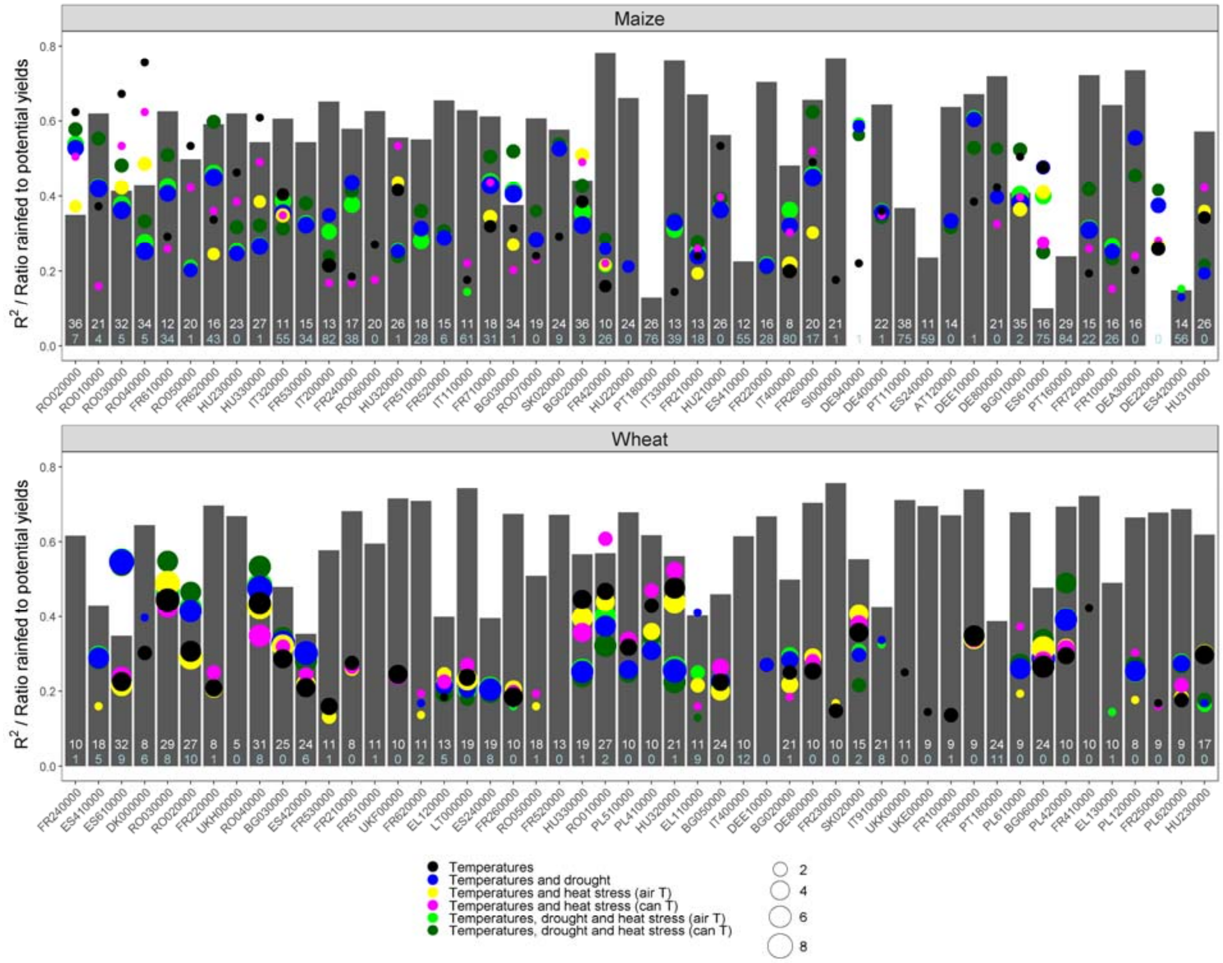

***Supplementary Figure 7*. Model skill and explained variation at NUTS2 level for grain maize and winter wheat.** *The coefficient of determination (R^2) between observed yields as reported in CAPRI database for the period between 1984 and 2009 and the six simulation sets (black - mean temperature effects only, blue – mean temperature and drought effects, yellow – mean temperature and heat stress with air-temperature effects, magenta - mean temperature and heat stress with canopy-temperature effects, light green - mean temperature, water-limitation and heat stress with air-temperature effects, and dark green - mean temperature, drought and heat stress with canopy-temperature effects). Each point represents the mean of the correlation coefficient for the eight winter wheat models and six maize models and the size of the dot indicates the number of models that had significant correlations for that simulation set and were considered in the respective mean. Grey columns serve as an environmental index indicating the model median average of rainfed to potential yields for each country. NUTS2 for the 50 NUTS2 with the largest production area are ordered by production area in descending order. Numbers in white at the base of the grey bars indicate the coefficient of variation in the observations. Numbers in light blue at the base of the grey bars indicate the ratio of irrigated to total maize production in the NUTS2 zone.*

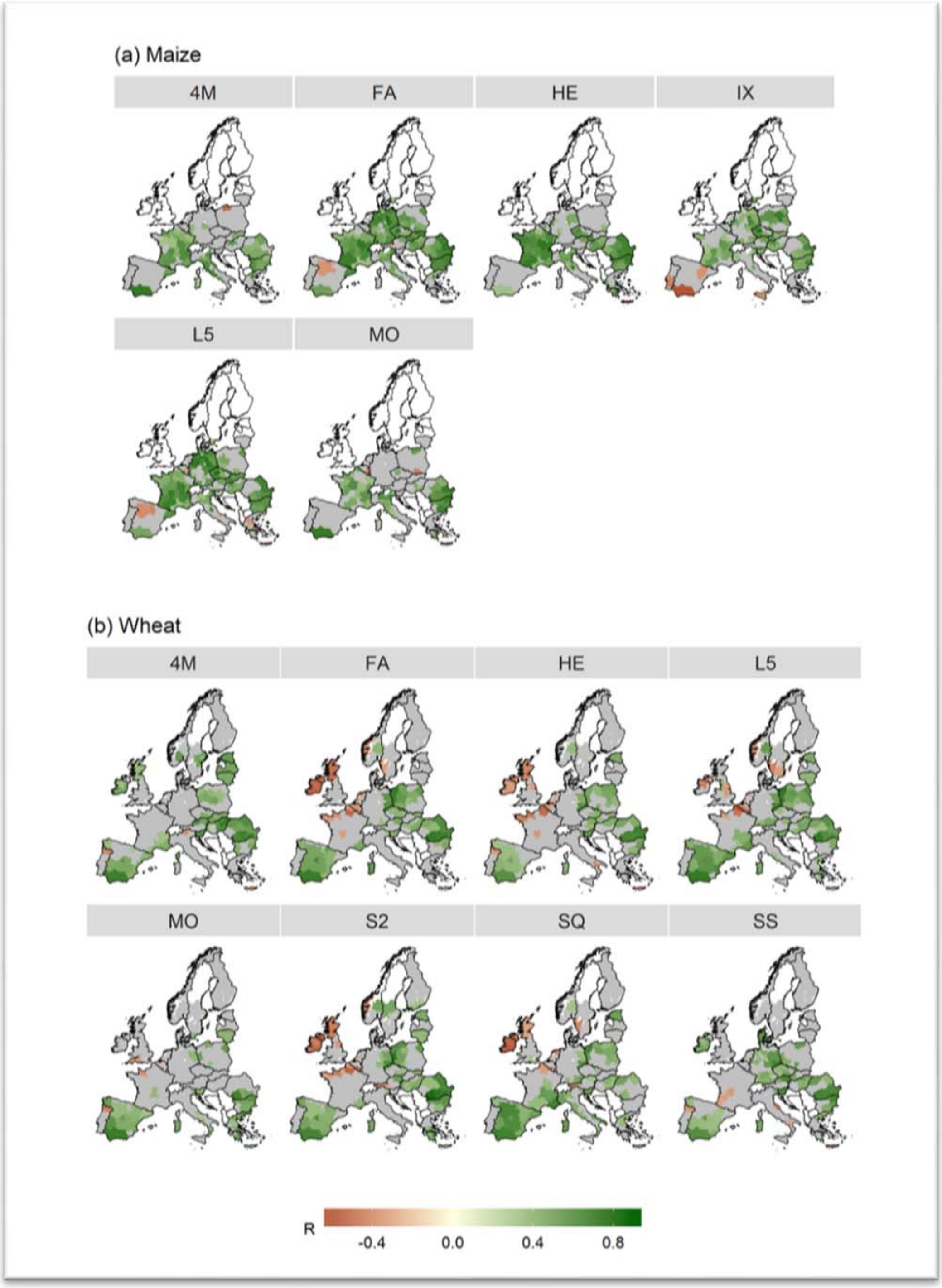

Supplementary Figure 8. *The correlation coefficient (R) between observed yields as reported at the NUTS2 sub-national administrative level for the period between 1986 and 2009 for the simulations (see Table S1 for names and details for each model) considering mean temperature, drought, and high air-temperature effects for (a) grain maize and (b) winter wheat*

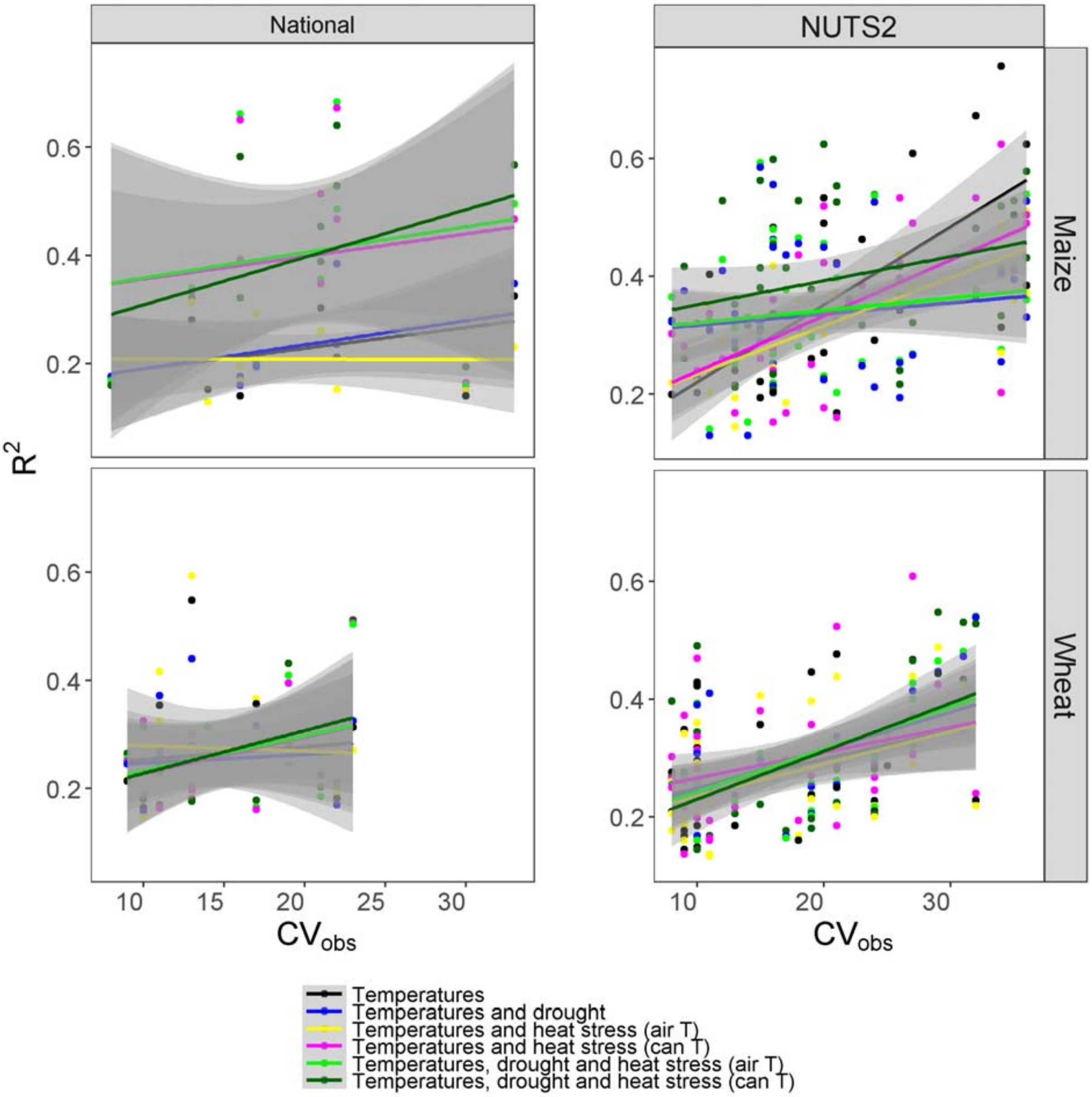

Supplementary Figure 9. Model skill and coefficient of variation of the observations (CV). *The relationship between model skill expressed as coefficient of determination (R^2) between observed yields and simulations and the coefficient of variation (CV) in the observed yield data at the national and NUTS2 level for grain maize and winter wheat. Analysis at the national level was for the period between 1981 and 2009 while analysis at the subnational NUTS2 level was for the period 1986 to 2009 for six simulation sets (black- mean temperature effects only, blue – mean temperature and drought effects, yellow – mean temperature and high air-temperature effects, magenta - mean temperature and high canopy-temperature effects, green - mean temperature, drought and high air-temperature effects, and dark green - mean temperature, drought and high canopy-temperature effects) over all models.*

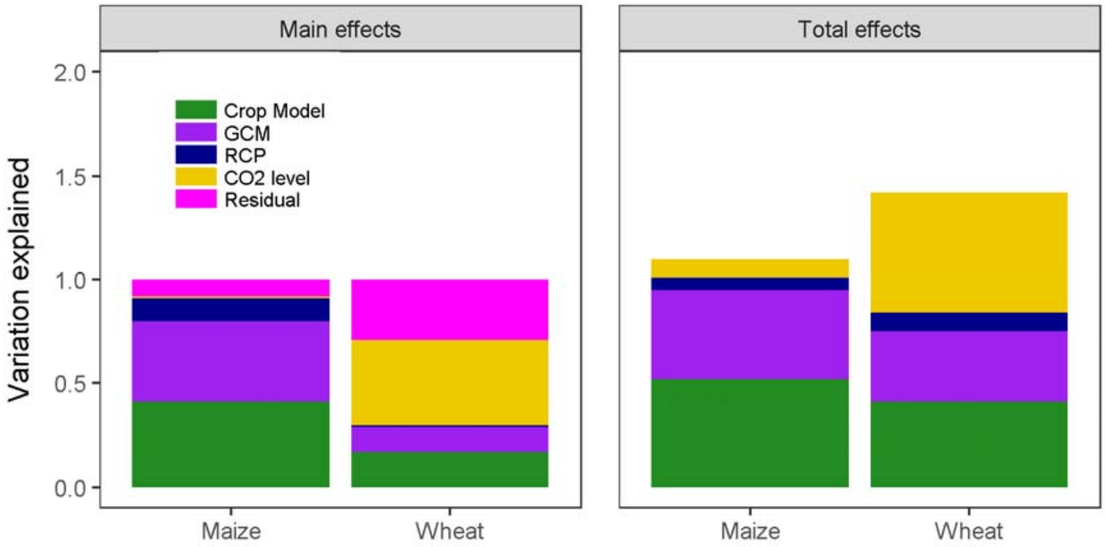

Supplementary Figure 10. *Main (first order) and total effects sensitivity indices of different sources of variation in the simulations of EU aggregate crop yields for current maize and wheat production areas to 2050. Sources of variation include: crop model (green), generalized circulation model (purple), representative concentration pathway (RCP, blue), [CO_2] level (gold) with the residual variation for the first order indices indicated by magenta.*

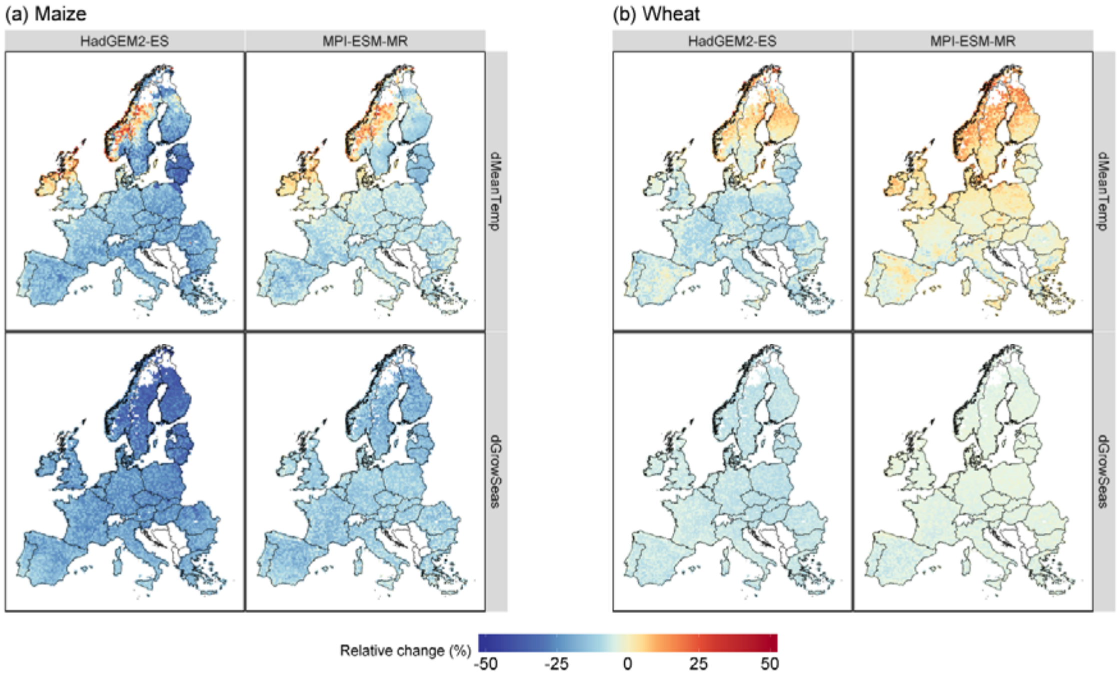

Supplementary Figure 11. *Relationship between of yield change due to mean temperature effects (dMeanTemp, top row) and the change in the length of the growing season (dGrowSeas, bottom row) for two general circulation models, HadGEM2-ES (first column in each panel) and MPI-ESM-RM (second column in each panel) to the period 2040 to 2069 relative to the baseline (1980 to 2010) for RCP4.5 for (a) grain maize and (b) winter wheat.*

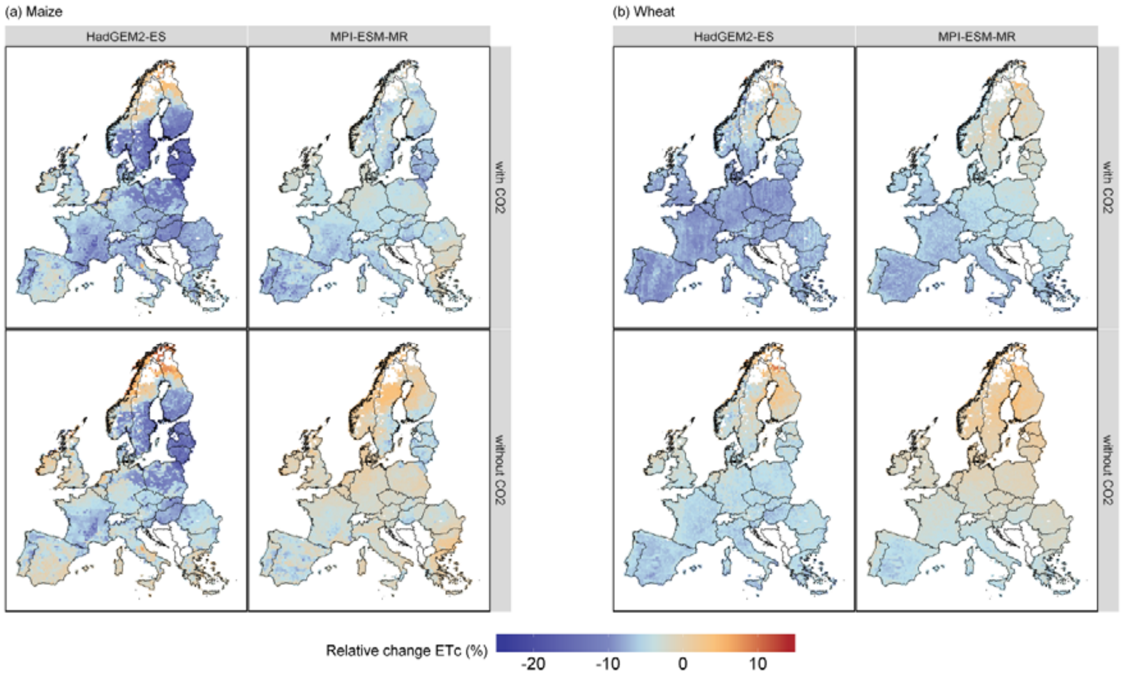

***Supplementary Figure* 12. Relative change in growing season crop water use (ET_c) for the period 2040 - 2069 relative to the baseline (1981 - 2010) for RCP 4.5 for current varieties and management of (a) grain maize and (b) winter wheat.** In each panel, simulations are shown with (top row) and without (bottom row) the effects of elevated [CO_2]. Results are shown for two generalized circulation models (GCMs) in each panel, HadGEM2-ES (first column) and MPI-ESM-RM (second column).

Supplementary Table 1. Description of the crop models and their consideration of CO_2 and water limitation effects.

MODEL	CROP	Effects of increasing [CO_2]			Water		
		Temperature	Transpiration	RUE	ET Method	Water stress	Evaporation/Transpiration distribution
FASSET (FA)	MZ, WW	Elevated CO_2 affects transpiration, which in turn affects daily maximum canopy temperature[1]	An empirical function reduces transpiration with elevated CO_2 [2]	An empirical function increases RUE with elevated CO_2 differentially for C3 and C4 crops [2]	Modified Makkink method[3]	Soil water stress is calculated as the ratio of actual to potential transpiration. The dry matter growth is proportional to this ratio. In addition, leaf senescence is enhanced when this ratio is lower than 1[4]	Potential ET is divided between crop and soil simulated LAI[4]
SIMPLACE (L5)	MZ, WW	Elevated CO_2 reduces stomatal conductance in non-water limited conditions which reduces canopy cooling for any given level of transpiration which results in higher canopy temperature (T_c)[1]	An empirical function reduces potential transpiration rates with elevated CO_2 differentially for C3 and C4 crops[5]	An empirical function increases RUE with elevated CO_2 differentially for C3 and C4 crops[5]	FAO-56 Penman-Monteith[6]	Soil water stress calculated as ratio of actual to potential transpiration. Used to reduce rates of biomass accumulation and leaf area expansion. Also increase partitioning of biomass to roots. Used in calculation of Tc between the upper (no transpiration) and lower (full transpiration) limit of Tc. The rate of transpiration also influences the energy balance of each limit as well as the stability correction terms.	Dual crop coefficient approach of FAO-56 [6]
HERMES (HE)	MZ, WW	When canopy temperature is used the reduced transpiration under elevated CO2 increase canopy temperature relative to the approach with air temperature	Standard stomata resistance in FAO-56 is replaced by a dynamic value derived by a function suggested by Yu et al.[7] depending on daily gross assimilation, CO_2 and vapor pressure deficit.	CO_2 effect on photosynthesis (P-R approach) is considered by a non-linear function according to Hoffmann[8] for C3, no effect for C4. Details are given in Kersebaum & Nendel[9]	FAO-56 Penman-Monteith[6]	Soil water stress calculated as ratio of actual to potential transpiration. Acceleration of crop development due to water stress. Reduced transpiration increase Tc.	One crop coefficient approach of FAO-56 [6]
MONICA (MO)	MZ, WW	None	Crop stomata resistance in FAO-56 model is calculated according to Yu et al.[7] depending on CO2	For photosynthesis of C3 crops, the dependency of maximum photosynthesis	FAO-56 Penman-Monteith[6] on the basis of crop LAI and	Drought stress of the crop is indicated by the relation of actual to potential transpiration, a reduction factor that acts directly on gross CO2 assimilation if crop-	Ground coverage determines to what extent transpiration contributes to total evapotranspiration

			concentration, daily gross assimilation, and vapor pressure deficit.	rate and light use efficiency to CO2 are described by non-linear functions proposed by Mitchell et al.[10] CO2 effects are not simulated for C4 crops	height, where potential ET is calculated from reference ET and developmental stage-specific Kc factors, minus interception storage	specific thresholds are exceeded.	
4M (4M)	MZ, WW	N/A	An empirical function reduces potential transpiration rates with elevated CO_2 differentially for C3 and C4 crops	An empirical function increases RUE with elevated CO_2 differentially for C3 and C4 crops	Priestley-Taylor method[11]	Soil water stress calculated as ratio of actual to potential transpiration. Used to (1) reduce rates of biomass accumulation, (2) accelerate leaf senescence	Potential E and T are partitioned based on simulated LAI. Suleiman and Ritchie[12] method for actual E based on upward flux calculations from all soil layers.
SSM (SS)	WW	Elevated CO_2 affects transpiration, thus canopy temperature through affecting the energy balance	An empirical function increases transpiration efficiency with elevated CO_2[13]	An empirical functions increases RUE with elevated CO_2 [13]	Priestley-Taylor method[11] as modified and described by Ritchie[14]	Water deficit hastens phenological development, reduces leaf expansion rate, and biomass accumulation [15]. A certain number of consecutive flooding days (20 d) results in crop death[15]	Soil evaporation using a two-stage evaporation model[16]. Daily transpiration rate is calculated using transpiration efficiency coefficient and vapor pressure deficit[16]
SiriusQuality version 3 (SQ)	WW	None	None	An empirical function increases RUE with elevated CO_2 [17]	Penman[18]	Soil water deficit calculated using a moisture deficit chocking function. Used to reduce leaf expansion rate, biomass accumulation, transpiration, and accelerate leaf senescence[19]	Soil evaporation calculated using Ritchie approach[18,20,21]
SIRIUS 2015 (S2)	WW	none	none	Increase of RUE with elevated CO_2[17]	Penman[18], but if wind and/or VP are not available, use Priestley-Taylor	Water stress reduces leaf expansion rate, biomass accumulation, transpiration, and accelerate leaf senescence	Soil evaporation calculated using Ritchie approach[14,18]
DSSAT_IX (IX)	MZ	None	Calculates a relative transpiration rate between current and elevated CO_2 conditions, affecting the leaf stomatal resistance and canopy resistance	Empirical C4 function increases potential growth rate, calculated from hourly leaf assimilation and daily canopy respiration, with elevated CO_2	FAO-56 Penman-Monteith[6]	Two water stresses based on the ratio of actual to potential T. The most limiting reduces expansion processes. The less limiting reduces growth processes.	Potential E and T are partitioned based on simulated LAI. Suleiman and Ritchie[12] method for actual E based on upflux calculations from all soil layers.

Supplementary Table 2. Description of the crop models and their approaches to simulating heat stress, canopy temperature and phenology.

MODEL	CROP	Heat stress response	Canopy temperature	Phenology approach	Temperature sensitivity of processes (not included as heat stress)
FASSET (FA)	MZ, WW	Heat stress affects leaf senescence enhancing leaf senescence rate when daily maximum temperature (Tc) exceeds a threshold of 30 C [22]	Based on an empirical relationship between midday crop temperature, evapotranspiration and net radiation[23]. Maximum and minimum Tc are calculated on a daily time step	Thermal time for maize and wheat with no heat stress effects. Wheat in addition considered photoperiod.	Phenology, leaf area expansion and leaf senescence, RUE
SIMPLACE (L5)	MZ, WW	The module reduces yield (Y) as a function of the hourly stress thermal time (TThs, in ◦Ch) accumulated above a critical high temperature threshold (Tcrit), being 31 or 34◦C (wheat or maize) This module is applied during the critical period for kernel number determination[24]	T_c is calculated from an **hourly** energy balance by summing incident solar radiation, soil, latent and sensible (*H*) heat fluxes and solving T_c from *H*. Atmospheric stability is considered by using Monin-Obukhov Similarity Theory (MOST) and empirical stability correction factors to solve for r_a. T_c is calculated for two bounding extremes: upper (no transpiration) and lower (full transpiration) limits of T_c, avoiding the need to specify canopy resistance terms at intermediate transpiration rates. With these two extreme potential values of T_c, actual $T_c = T_{c,L} + (1 - K_{WS})(T_{c,U} - T_{c,L})$ where K_{ws} is soil water stress index. A full description is given by Webber et al[25]	Thermal time for maize and wheat with no heat stress effects, Wheat additionally considers photoperiod and vernalization	phenology, leaf area expansion in juvenile phase (before driven by biomass accumulation and specific leaf area), RUE and evapotranspiration
HERMES (HE)	MZ, WW	Approach acc. to MONICA: Daily temperatures above a threshold during 10 days from flowering create a descending stress factor which is accumulated during the 10 days. The final factor is applied to biomass increase of grains during the rest of the season.	Tc is calculated from an hourly energy balance by summing incident solar radiation, latent and sensible (H) heat fluxes and solving Tc from H. Hourly temperature and radiation values are determined following Hoogenboom & Huck[26]. The sensible heat flux is given by: H=(ρc_P (T_c-T_a))/r_a , where ρ is air density, cp the specific heat of air, Ta is air temperature and ra is the aerodynamic resistance which is calculated according to Thom and Oliver[27] as ra= [4.72[ln ((z - d + zo)/zo)] 2]/(1 + 0.54u) where u is wind speed at reference height z, d the zero-displacement height equal 1.04h0.88 and zo the roughness length for momentum and heat transfer each equal to z0 = 0.062h1.08, where h is the crop height.	Thermal time for maize and wheat with no heat stress effects, Wheat additionally considers photoperiod and vernalization	Specific temperature functions for C3 and C4 crops for gross photosynthesis, and temperature dependent respiration. Net assimilation (P-R) is strongly affected by temperature.
MONICA (MO)	MZ, WW	A sensitive phase for heat stress is defined around flowering. In this period, temperatures above 31°C for wheat or 35°C for maize determine a linear increment of	None	Thermal time for maize and wheat. Water stress after flowering accelerates development. Wheat additionally considers	Phenology, photosynthesis and respiration, evapotranspiration, root growth, soil organic matter turnover, soil nitrogen processes

		a stress factor [28] applied to the fraction of opened flowers [29]. The cumulative stress factor reduces the partitioning of assimilates to the grains.		photoperiod and vernalization	
4M (4M)	MZ, WW	Incomplete pollination as a function of Tmax. Number of kernels available for grain filling is reduced above 30 °C (wheat) and 35 °C (maize) in the flowering stage	N/A	Thermal time for maize and wheat with no heat stress effects, Wheat additionally considers photoperiod and vernalization	Phenology, photosynthesis and respiration, evapotranspiration, leaf senescence, soil nitrogen processes
SSM (SS)	WW	Maximum daily temperatures above a threshold value (30°C) reduce leaf expansion and accelerate leaf senescence. The daily seed growth rate is progressively reduced by temperatures higher than 31°C and is null when temperature is greater than 40°C	T_c is calculated from a **daily** energy balance assuming neutral atmospheric stability (Jamieson *et al.*, 1995).	Phenological development is calculated based on the biological day concept. A biological day is a day with optimal temperature, photoperiod and moisture conditions for plant development[15]	Phenology (including leaf appearance rate and vernalization), leaf area expansion, radiation use efficiency, evapotranspiration[15]
SIRIUS QUALITY (SQ)	WW	Leaf senescence increases linearly above a threshold value (31°C) of maximum daily T_c [30]	T_c is calculated from a daily energy balance assuming neutral atmospheric stability [18]	Flowering time is calculated from the final number response to temperature and daylength[31,32]	Phenology (leaf appearance rate and vernalization), leaf expansion, biomass accumulation, biomass and nitrogen remobilization, evapotranspiration, soil nitrogen mineralization [33]
SIRIUS 2000 (S2)	WW	Decrease in grain number and grain size, accelerated leaf senescence during grain filling[34]	T_c is calculated from a daily energy balance assuming neutral atmospheric stability [18]	Anthesis is calculated from the final leaf number as a function of temperature and daylength[31]	Phenology, leaf expansion and senescence, RUE, biomass accumulation, biomass and nitrogen remobilization, evapotranspiration, soil nitrogen mineralization
DSSAT_IX (IX)	MZ	Cohorts of plants reaching anthesis and shedding pollen and cohorts of plants exposing silks separated by ASI days. Hourly temperatures extrapolated from Tmax and Tmin are scanned and compared against a critical Tc (35 °C) and a sterilizing Ts (41 °C). Number of hours above Tc and Ts reduce daily the number of exposed silks and the potential kernel set[35]	Not considered	Thermal time calculated with the simplified beta function[36] parametrized for vegetative and reproductive phases	Temperature functions affecting leaf assimilation, canopy respiration, leaf expansion, and grain growth rate

***Supplementary* Table 3: Significance of main treatment effects and interactions from statistical analyses of relative change to 2050 in European yield levels aggregated by current areas for rainfed and irrigated production.** Results are shown for each of the: three-way fixed effects ANOVA on means (3-fixed-ANOVA), three-way mixed model ANOVA on means (3-mixed-ANOVA), three-way mixed model ANOVA on means with crop model and GCM as random factors (3-mixed-ANOVA-rModGCM), three-way mixed model ANOVA on means with GCM nested in crop model as random factors (3-mixed-ANOVA-rModNestGCM) and two-way fixed test on median (2-way-median).

**Effect**	**3-fixed-ANOVA**	**3-mixed-ANOVA**	**3-mixed-ANOVA-rModGCM**	**3-mixed-ANOVA-rModNestGCM**	**2-way-median**
Crop	***	***	***	***	***
CO_2	***	***	***	***	***
Scenario	**	**	***	***	NA
Crop x CO_2	***	***	***	***	**
Crop x Scenario	*	**	***	***	NA
CO_2 x Scenario	*	*	***	***	NA
Crop x CO_2 x Scenario	ns	ns	ns	ns	NA

* Indicates significant at $p<0.05$
** Indicates significant at $p< 0.01$
*** Indicates significant at $p<0.001$
ns indicates non-significant
NA not tested

***Supplementary* Table 4: Significance of main treatment effects and interactions from statistical analyses on relative yield losses due to drought to 2050 in European yield levels aggregated by current areas for rainfed production.** Results are shown for each of the: three-way fixed effects ANOVA on means (3-fixed-ANOVA), three-way mixed model ANOVA on means (3-mixed-ANOVA), three-way mixed model ANOVA on means with crop model and GCM as random factors (3-mixed-ANOVA-rModGCM), three-way mixed model ANOVA on means with GCM nested in crop model as random factors (3-mixed-ANOVA-rModNestGCM) and two-way fixed test on median (2-way-median).

**Effect**	**3-fixed-ANOVA**	**3-mixed-ANOVA**	**3-mixed-ANOVA-rModGCM**	**3-mixed-ANOVA-rModNestGCM**	**2-way-median**
Crop	***	***	***	***	***
CO_2	*	*	***	***	*
Scenario	ns	ns	**	**	NA
Crop x CO_2	ns	ns	**	**	ns
Crop x Scenario	ns	ns	ns	*	NA
CO_2 x Scenario	ns	ns	ns	ns	NA
Crop x CO_2 x Scenario	ns	ns	ns	ns	NA

* Indicates significant at p<0.05
** Indicates significant at p< 0.01
*** Indicates significant at p<0.001
ns indicates non-significant
NA not tested

***Supplementary* Table 5: Significance of main treatment effects and interactions from statistical analyses on relative yield losses due to heat to 2050 in European yield levels aggregated by current areas for rainfed production.** Results are shown for each of the three-way fixed effects ANOVA on means (3-fixed-ANOVA), and two-way fixed test on median (2-way-median).

**Effect**	**3-fixed-ANOVA**	**2-way-median**
Crop	**	ns
CO_2	ns	ns
Scenario	ns	NA
Crop x CO_2	ns	ns
Crop x Scenario	ns	NA
CO_2 x Scenario	ns	NA
Crop x CO_2 x Scenario	ns	NA

* Indicates significant at p<0.05
** Indicates significant at p< 0.01
*** Indicates significant at p<0.001
ns indicates non-significant
NA not tested

***Supplementary* Table 6: Significance of main treatment effects and interactions from statistical analyses on absolute increase in low-yielding years as compared to average conditions in yield losses due to different drivers (mean temperature effects, drought or heat stress effects) to 2050 in European yields aggregated by current areas for rainfed production.** Results are shown for each of the: three-way fixed effects ANOVA on means (3-fixed-ANOVA), three-way mixed model ANOVA on means (3-mixed-ANOVA), three-way mixed model ANOVA on means with crop model and GCM as random factors (3-mixed-ANOVA-rModGCM), three-way mixed model ANOVA on means with GCM nested in crop model as random factors (3-mixed-ANOVA-rModNestGCM) and two-way fixed test on median (2-way-median Crop x CO_2).

**Effect**	**3-fixed-ANOVA**	**3-mixed-ANOVA**	**3-mixed-ANOVA-rModGCM**	**3-mixed-ANOVA-rModNestGCM**	**2-way-median (Crop x CO_2)**
Driver	***	***	***	***	NA
Crop	***	***	***	***	*
CO_2	ns	ns	ns	ns	ns
Scenario	ns	ns	ns	ns	NA
Driver x Crop	***	***	***	***	NA
Driver x CO2	ns	ns	ns	ns	NA
Driver x Scenario	ns	ns	ns	ns	NA
Crop x CO_2	ns	ns	ns	ns	ns
Crop x Scenario	ns	ns	ns	ns	NA
CO_2 x Scenario	ns	ns	ns	ns	NA
Driver x Crop x CO_2	ns	ns	ns	ns	NA
Driver x Crop x Scenario	ns	ns	ns	ns	NA
Driver x CO_2 x Scenario	ns	ns	ns	ns	NA
Crop x CO_2 x Scenario	ns	ns	ns	ns	NA
Driver x Crop x CO_2 x Scenario	ns	ns	ns	ns	NA

* Indicates significant at $p<0.05$
** Indicates significant at $p< 0.01$
*** Indicates significant at $p<0.001$
ns indicates non-significant
NA not tested

Supplementary Methods

The details of the modelling protocol followed to generate the simulation results are detailed below:

1. **Data access**
   Access to all input data is available by emailing Heidi Webber at: Heidi.webber@mail.mcgill.ca (permanent address)
2. **Study extent**
   The study is conducted over the EU-27 for the 8157 grid cells with all data inputs have been prepared for use with these grids. The final selection of grid cells for simulation in this study is less than the total number of climate grids (8709). The extent of the current study is based on selecting climate grids in which there was current agricultural land use (2006 Corine Land Use Map v17) and aggregate soil depth of at least 40 cm
3. **Climate data description**
   All climate data (all periods, and RCM_RCP combinations) for a simulation grid (total of 8709) are contained in a single file (name indicates the grid, row_col), compressed with gzip. To avoid issues with leap years, all scenario climate data use dates from the 1980 - 2010 period. To distinguish the time periods from one another, please use the field "period" (0 = 1980-2010; 2 = 2040-2069; 3 =2070-2099)
4. **Soil data and initial soil moisture data**
   Soil data, including the assumed initial soil moisture, for all simulation grids are saved in a single csv file. Please consider a maximum root depth as 1.5 m (both wheat and maize) or the depth of soil, if less than 1.5 m.
5. **Phenology observations**
   Observed sowing dates, anthesis dates and harvest/maturity dates for all simulation grids are saved in a one csv file for each of maize and winter wheat. The original observations for sowing, anthesis and harvest for winter wheat and maize were taken from Eurostat (http://ec.europa.eu/eurostat/web/main). Across Europe, approx. 220000 observations for the period 1928-2006 were aggregated to 13 environmental zones (FIRST_ENZ field in the shapefile) [37]. The values in the environmental zones were further disaggregated and assigned to the grid cells.**Starting simulations and re-initialization**
   ASimulations are started on the day of (or 1 day before, if required) the reported mean sowing day for each grid, reinitializing each year, for all simulation periods and scenarios
6. **Calibration**
   Only observed antheisis and harvest dates are be calibrated. Using the historical climate data (period = 0) and the phenology observations, for each grid cell, crop thermal times (and associated vernalization and photoperiod parameters) are selected to match (averaged over the 1980-2010 period) observed and simulated anthesis and maturity dates. The calibrated thermal times (and other phenology parameters) are used for all scenario simulations.
7. **Simulation steps**
   For each grid (8157), simulations are performed for both crops (grain maize= "Maize" and winter wheat= "WW"), six simulation treatments as indicated below, for each of the 48 gcm_rcp-period-CO2 combinations defined below. Model that do not simulate canopy temperature, do not simulate treatments T3 or T6.

Simulation treatments with respective codes for TrtNo, Irrigation status and Production case to be used for reporting results

TrtNo	Irrigation status (code)	Production case (code)	How to accomplish
T1	Full	Potential (Pot)	switch off heat stress (ie, set threshold temperature at 70°C)
T2	Full	Heat-limited with air temperature (HL_air)	use air temperature as input to heat stress routines, but keep all other processes (RUE, dev rate, photosynthesis) using regular temperature inputs
T3	Full	Heat-limited with canopy temperature (HL_can)	use canopy temperature as input to heat stress routines, but keep all other processes (RUE, dev rate, photosynthesis) using regular temperature inputs
T4	Rain	Water limited with no heat stress (WL)	switch off heat stress (ie, set threshold temperature at 70°C)
T5	Rain	Water-heat-limited with air temperature (WHL_air)	use air temperature as input to heat stress routines, but keep all other processes (RUE, dev rate, photosynthesis) using regular temperature inputs
T6	Rain	Water - heat- limited with canopy temperature (WHL_can)	use canopy temperature as input to heat stress routines, but keep all other processes (RUE, dev rate, photosynthesis) using regular temperature inputs

Unique identifiers for the 48 period * gcm_rcp * CO_2 levels for which simulations are conducted

ClimPerCO2_ID	period	gcm_rcp	CO2	ClimPerCO2_ID	period	gcm_rcp	CO2
C1	0	0_0	360	-	-	-	-
C2	2	GFDL-CM3_45	360	C26	2	GFDL-CM3_45	499
C3	3	GFDL-CM3_45	360	C27	3	GFDL-CM3_45	532
C4	2	GFDL-CM3_85	360	C28	2	GFDL-CM3_85	571
C5	3	GFDL-CM3_85	360	C29	3	GFDL-CM3_85	801
C6	2	GISS-E2-R_45	360	C30	2	GISS-E2-R_45	499
C7	3	GISS-E2-R_45	360	C31	3	GISS-E2-R_45	532
C8	2	GISS-E2-R_85	360	C32	2	GISS-E2-R_85	571
C9	3	GISS-E2-R_85	360	C33	3	GISS-E2-R_85	801
C10	2	HadGEM2-ES_26	360	C34	2	HadGEM2-ES_26	442
C11	3	HadGEM2-ES_26	360	C35	3	HadGEM2-ES_26	429
C12	2	HadGEM2-ES_45	360	C36	2	HadGEM2-ES_45	499
C13	3	HadGEM2-ES_45	360	C37	3	HadGEM2-ES_45	532
C14	2	HadGEM2-ES_85	360	C38	2	HadGEM2-ES_85	571
C15	3	HadGEM2-ES_85	360	C39	3	HadGEM2-ES_85	801
C16	2	MIROC5_45	360	C40	2	MIROC5_45	499
C17	3	MIROC5_45	360	C41	3	MIROC5_45	532
C18	2	MIROC5_85	360	C42	2	MIROC5_85	571
C19	3	MIROC5_85	360	C43	3	MIROC5_85	801
C20	2	MPI-ESM-MR_26	360	C44	2	MPI-ESM-MR_26	442
C21	3	MPI-ESM-MR_26	360	C45	3	MPI-ESM-MR_26	429
C22	2	MPI-ESM-MR_45	360	C46	2	MPI-ESM-MR_45	499
C23	3	MPI-ESM-MR_45	360	C47	3	MPI-ESM-MR_45	532
C24	2	MPI-ESM-MR_85	360	C48	2	MPI-ESM-MR_85	571
C25	3	MPI-ESM-MR_85	360	C49	3	MPI-ESM-MR_85	801

8. **Prepare outputs**

One output file per simulation grid, for a total of 8157 output files. All period by gcm_rcp by CO_2 levels, treatments and crops for a grid cell will be in the same file.

Each output file has: 2 crops (Maize or WW); 49 climate by period by CO_2 combinations (ClimPerCO2_ID); 6 treatments (TrtNo) and 30 years (harvest year, ie 1981 to 2010, do not output 1980 for maize), for a total of 17 640 lines of output data (excluding header) for each of the 8157 files
with any missing values reported as "na"

9. **Naming, saving and sending your outputs** (8157 compressed files)
   Each output file is named as: "EU_HS_Your2digitModelCode_row_col.csv.gz (e.g. for the STICS model, on grid 54_119 would result in a file: EU_HS_ST_54_119.txt)
   The 2-digit code for each model is listed below.
   …

2-digit model for naming files and output folders

**Model (code)**	**Model 2-letter code**
HERMES	HE
Simplace<Lintul5, Slim3, FAO-56 ET0>	L5
Nwheat	AN
SiriusQuality	SQ
MONICA	MO
Sirius2014	S2
FASSET	FA
4M	4M
SSM	SS
DSSAT-CSM Ixim & DSSAT CERES (control)	IX

References

1 Webber, H. *et al.* Physical robustness of canopy temperature models for crop heat stress simulation across environments and production conditions. *Field Crops Research* **216**, 75 - 88 (2018).

2 Doltra, J., Lægdsmand, M. & Olesen, J. E. Impacts of projected climate change on productivity and nitrogen leaching of crop rotations in arable and pig farming systems in Denmark. *The Journal of Agricultural Science* **152**, 75-92 (2014).

3 Hansen, S. Estimation of Potential and Actual EvapotranspirationPaper presented at the Nordic Hydrological Conference (Nyborg, Denmark, August-1984). *Hydrology Research* **15**, 205-212 (1984).

4 Olesen, J. E. *et al.* Comparison of methods for simulating effects of nitrogen on green area index and dry matter growth in winter wheat. *Field Crops Research* **74**, 131-149 (2002).

5 Zhao, G. *et al.* The implication of irrigation in climate change impact assessment: a European wide study. *Global Change Biology* **21**, 4031–4048, doi:10.1111/gcb.13008 (2015).

6 Allen, R. G., Pereira, L. S., Raes, D. & Smith, M. Crop evapotranspiration-Guidelines for computing crop water requirements-FAO Irrigation and drainage paper 56. *FAO, Rome* **300**, 6541 (1998).

7 Yu, O., Goudriaan, J. & Wang, T.-D. Modelling Diurnal Courses of Photosynthesis and Transpiration of Leaves on the Basis of Stomatal and Non-Stomatal Responses, Including Photoinhibition. *Photosynthetica* **39**, 43-51, doi:10.1023/a:1012435717205 (2001).

8 Hoffmann, F. FAGUS, a model for growth and development of beech. *Ecological Modelling* **83**, 327-348 (1995).

9 Kersebaum, K. & Nendel, C. Site-specific impacts of climate change on wheat production across regions of Germany using different CO 2 response functions. *European Journal of Agronomy* **52**, 22-32 (2014).

10 Mitchell, R. *et al.* Effects of elevated CO2 concentration and increased temperature on winter wheat: test of ARCWHEAT1 simulation model. *Plant, Cell & Environment* **18**, 736-748 (1995).

11 Priestley, C. & Taylor, R. On the assessment of surface heat flux and evaporation using large-scale parameters. *Monthly weather review* **100**, 81-92 (1972).

12 Suleiman, A. A. & Ritchie, J. T. Modifications to the DSSAT vertical drainage model for more accurate soil water dynamics estimation. *Soil science* **169**, 745-757 (2004).

13 Ludwig, F. & Asseng, S. Climate change impacts on wheat production in a Mediterranean environment in Western Australia. *Agricultural Systems* **90**, 159-179 (2006).

14 Ritchie, J. in *Understanding options for agricultural production* 41-54 (Springer, 1998).

15 Soltani, A., Maddah, V. & Sinclair, T. SSM-Wheat: a simulation model for wheat development, growth and yield. *International Journal of Plant Production* **7**, 711-740 (2013).

16 Soltani, A. *Modeling physiology of crop development, growth and yield*. (CABi, 2012).

17 Jamieson, P. D. *et al.* Modelling CO2 effects on wheat with varying nitrogen supplies. *Agriculture, ecosystems & environment* **82**, 27-37 (2000).

18 Jamieson, P., Brooking, I., Porter, J. & Wilson, D. Prediction of leaf appearance in wheat: a question of temperature. *Field Crops Research* **41**, 35-44 (1995).

19 Martre, P. *et al.* Modelling protein content and composition in relation to crop nitrogen dynamics for wheat. *European Journal of Agronomy* **25**, 138-154 (2006).

20 Ritchie, J. T. Model for predicting evaporation from a row crop with incomplete cover. *Water resources research* **8**, 1204-1213 (1972).

21 Tanner, C. & Jury, W. Estimating Evaporation and Transpiration from a Row Crop during Incomplete Cover 1. *Agronomy Journal* **68**, 239-243 (1976).

22 Vignjevic, M., Wang, X., Olesen, J. E. & Wollenweber, B. Traits in spring wheat cultivars associated with yield loss caused by a heat stress episode after anthesis. *Journal of Agronomy and Crop Science* **201**, 32-48 (2015).
23 SEGUIN, B. & ITIER, B. Using midday surface temperature to estimate daily evaporation from satellite thermal IR data. *International Journal of Remote Sensing* **4**, 371-383 (1983).
24 Gabaldón-Leal, C. *et al.* Modelling the impact of heat stress on maize yield formation. *Field Crops Research* **198**, 226-237 (2016).
25 Webber, H. A. *et al.* Simulating canopy temperature for modelling heat stress in cereals. *Environmental Modelling & Software* **77**, 143-155 (2016).
26 Hoogenboom, G. & Huck, M. G. Rootsimu v4. 0: A dynamic simulation of root growth, water uptake, and biomass partitioning in a soil-plant-atmosphere continuum: Update and documentation. *Agronomy and soils departmental series-Auburn University, Alabama Agricultural Experiment Station (USA)* (1986).
27 Thom, A. & Oliver, H. On Penman's equation for estimating regional evaporation. *Quarterly Journal of the Royal Meteorological Society* **103**, 345-357 (1977).
28 Challinor, A., Wheeler, T., Craufurd, P. & Slingo, J. Simulation of the impact of high temperature stress on annual crop yields. *Agricultural and Forest Meteorology* **135**, 180-189 (2005).
29 Moriondo, M., Giannakopoulos, C. & Bindi, M. Climate change impact assessment: the role of climate extremes in crop yield simulation. *Climatic Change* **104**, 679-701 (2011).
30 Maiorano, A. *et al.* Crop model improvement reduces the uncertainty of the response to temperature of multi-model ensembles. *Field Crops Research* **202**, 5–20 (2017).
31 Jamieson, P. D. *et al.* A comparison of the models AFRCWHEAT2, CERES-wheat, Sirius, SUCROS2 and SWHEAT with measurements from wheat grown under drought. *Field Crops Research* **55**, 23-44 (1998).
32 He, J. *et al.* Simulation of environmental and genotypic variations of final leaf number and anthesis date for wheat. *European Journal of Agronomy* **42**, 22-33 (2012).
33 Wang, E. *et al.* The uncertainty of crop yield projections is reduced by improved temperature response functions. *Nature plants* **3**, 17102 (2017).
34 Stratonovitch, P. & Semenov, M. A. Heat tolerance around flowering in wheat identified as a key trait for increased yield potential in Europe under climate change. *Journal of experimental botany*, erv070 (2015).
35 Lizaso, J. *et al.* Modeling the response of maize phenology, kernel set, and yield components to heat stress and heat shock with CSM-IXIM. *Field Crops Research* **214**, 239-252 (2017).
36 Metzger, M., Bunce, R., Jongman, R., Mücher, C. & Watkins, J. A climatic stratification of the environment of Europe. *Global Ecology and Biogeography* **14**, 549-563 (2005).
